# Revisiting the Development of Vaccines Against Pathogenic *Leptospira*: Innovative Approaches, Present Challenges, and Future Perspectives

**DOI:** 10.3389/fimmu.2021.760291

**Published:** 2022-01-03

**Authors:** Giovana C. Barazzone, Aline F. Teixeira, Bruna O. P. Azevedo, Deborah K. Damiano, Marcos P. Oliveira, Ana L. T. O. Nascimento, Alexandre P. Y. Lopes

**Affiliations:** ^1^ Laboratório de Desenvolvimento de Vacinas, Instituto Butantan, São Paulo, Brazil; ^2^ Programa de Pós-Graduação Interunidades em Biotecnologia, Instituto de Ciências Biomédicas, Universidade de São Paulo (USP), São Paulo, Brazil

**Keywords:** *Leptospira*, vaccine, leptospirosis, human leptospirosis vaccine, development of vaccine

## Abstract

Human vaccination against leptospirosis has been relatively unsuccessful in clinical applications despite an expressive amount of vaccine candidates has been tested over years of research. Pathogenic *Leptospira* encompass a great number of serovars, most of which do not cross-react, and there has been a lack of genetic tools for many years. These obstacles have hampered the understanding of the bacteria’s biology and, consequently, the identification of an effective antigen. Thus far, many approaches have been used in an attempt to find a cost-effective and broad-spectrum protective antigen(s) against the disease. In this extensive review, we discuss several strategies that have been used to develop an effective vaccine against leptospirosis, starting with *Leptospira*-inactivated bacterin, proteins identified in the genome sequences of pathogenic *Leptospira*, including reverse vaccinology, plasmid DNA, live vaccines, chimeric multi-epitope, and toll- and nod-like receptors agonists. This overview should be able to guide scientists working in the field to select potential antigens and to choose the appropriate formulation to administer the candidates.

## Introduction

Leptospirosis is a worldwide emerging zoonotic disease caused by pathogenic species of the genus *Leptospira*. Pathogenic leptospires colonize the proximal renal tubules of reservoir hosts that keep shedding live bacteria into the urine, contaminating the ecosystem. Humans are accidental and terminal hosts in the transmission of leptospirosis. They are infected directly through contact with urine or indirectly *via* contaminated soil or water (1). The symptoms of the disease are non-specific and flu-like: fever, chills, headache, nausea, vomiting, cough and diarrhea. Patients may evolve to severe conditions such as Weil’s syndrome, which has a mortality rate of up to 50%, or severe pulmonary hemorrhage syndrome (SPHS), where fatality rate can be superior to 70% (2). These syndromes are characterized by systemic complications, comprising jaundice, meningitis, pulmonary hemorrhage, hepatic and renal dysfunction, and cardiovascular collapse (1, 3).

After years of research, we still do not have a universal vaccine for leptospirosis. It has been considered a great challenge, since *Leptospira* was shown to be a quite complex pathogen. Nonetheless, it is an important goal to be achieved, since inactivated whole cells, also called bacterins, are the only vaccines commercially available, primarily for veterinary use. One of the main drawbacks of these vaccines is that the immunity elicited is mainly serovar dependent and based on lipopolysaccharide antigens. There are more than 300 classified serovars (4, 5). Epidemiologic studies are constantly necessary to verify serogroup prevalence. Moreover, inactivated vaccines do not promote long-term protection, where booster doses are necessary annually, and some side effects have been reported.

Some countries, including France, Cuba, China and Japan, have bacterin-like vaccines currently licensed for use in people in a high-risk occupation in contact with contaminated water or animals (6, 7). The first anti-leptospirosis vaccine was used in Japan in 1919 (8). A monovalent inactivated vaccine, Spirolept^®^, consisting of *L. interrogans* serogroup Icterohaemorrhagiae, the prevalent serogroup in France, has been available for specific groups since 1979. A clinical trial evaluating the administration route (subcutaneous or intramuscular) showed that a local reaction after vaccinations was more frequent with the subcutaneous route. The safety and immunogenicity of the vaccine were suitable for both cases. The importance of boosting this vaccine was demonstrated by monitoring antibody levels after a second booster injection 30 months after primary schedule vaccination (9).

Vax-Spiral^®^ was developed by Institute Finlay – Cuba, registered in 1998, and is composed of 50 – 80 x 10^6^ cells of serogroup Canicola serovar Canicola, serogroup Icterohaemorrhagiae serovar Copenhageni and serogroup Pomona serovar Mozdok adsorbed to alum. The phase III clinical trial showed vaccine efficacy of 78.1% and no serious adverse effects (10).

According to Xu and Ye (2018), leptospirosis incidence in China has been decreasing due to improvements in sanitation and vaccination of high-risk populations. However, leptospirosis remains endemic with localized outbreaks. As China is a huge country with a great variety of geographic and climatic conditions, the existing serovars are more diverse and epidemiological studies are challenging. Currently, a polyvalent inactivated vaccine composed of serogroups Icterohaemorrhagiae serovar Lai, Grippotyphosa serovar Linhai, Autumnalis serovar Autumnalis, Canicola serovar Canicola, Pomona serovar Pomona, Australis serovar Australis and Hebdomadis serovar Hebdomadis, in which the leptospires are grown in protein-free synthetic media, is available. This vaccine covers more than 80% of circulating serogroups and is recommended for high-risk populations during annual epidemic periods (7).

Besides the drawbacks of the inactivated whole-cell vaccines, such as the lack of immunological memory, reactogenicity issues and protection conferred primarily against the serovars included in the formulation, they are the only available option for humans. For animals, protection and sterilizing immunity are ideal, since these animals may have chronical and asymptomatic disease, shedding live leptospires. In the case of humans, which have mild or severe cases of the disease, protection would be the main goal.

## Whole-Genome Sequencing and Reverse Vaccinology

The advent of whole-genome sequencing has had an impressive impact on the microbial field. Genome sequences associated with bioinformatics tools has opened a new window of opportunities to search for antigen candidates against leptospirosis. Reverse vaccinology is a strategy based on the knowledge of the organism’s genome, high-throughput bioinformatics techniques and data analysis processes that allow the search for potential vaccine antigens. It is a strategy alternative to conventional vaccinology, which involves organism cultivation and antigen isolation and testing, one by one, until the finding and characterization of good vaccine candidates (11). Although, many vaccines were developed by using conventional methods, its use has been increasingly limited, especially for those pathogens that show a huge antigenic diversity. Reverse vaccinology allows finding vaccine candidates quickly and efficiently. One of the main advantages is the identification of proteins independently of their amount and the avoidance of growing the microorganism *in vitro* (12–14). The successful development of 4CMenB (Bexsero^®^, GSK) shows the feasibility of reverse vaccinology use. It was the first commercially approved human vaccine based on this approach. The vaccine is composed of *Neisseria* adhesin type A (NadA), neisserial heparin-binding antigen (NHBA), variant 1 of factor H binding protein (fHbp) protein, and the outer membrane vesicle (OMV) of *N. meningitidis* strain NZ98/254, a New Zealand outbreak strain (15, 16). The prediction of potential antigens *in silico* based on bioinformatics has narrowed down the universe to be studied.

Although *Leptospira* were identified about 100 years ago in Japan and Europe (4), leptospiral genome sequencing was only launched in the early years of the 21^st^ century and since then has made a remarkable contribution to the knowledge of bacterial biology, providing new insights into leptospirosis virulence and pathogenicity. Up to now, 30 new *Leptospira* species were identified and a new classification have been proposed as shown by Vincent et al. (5). In 2003, the *L. interrogans* serovar Lai whole-genome sequence was published and 4.7 mega base pairs were determined, distributed in two chromosomes (17). Among those, only 44% coding sequences (CDS) had their function assigned, whereas 41% had no function or similarity identified when compared to other sequences present in GenBank, including the spirochetes *Treponema pallidum* and *Borrelia burgdorferi*. One year later, *L. interrogans* serovar Copenhageni, strain Fiocruz L1-130, isolated from an outbreak in Brazil, had its genome sequenced. It was composed of 4.6 mega base pairs distributed in two chromosomes like serovar Lai, where 3,728 CDS were described, of which 1,972 had their function identified and 1,756 were hypothetical. Several surface proteins were identified and new ones were found as well (18, 19). Genome sequencing of *L. borgpetersenii* was completed like previously reported, adding more data to pathogenicity studies (20). Whole-genome sequencing of *L. biflexa* was reported in 2008 and provided new inputs into comparative studies between saprophytic and pathogenic strains (21). The major difference found was the lack of genes encoding virulence factors such as the Lig proteins and a putative factor H binding protein (LfhA).

Since genome sequencing, many studies have been performed to evaluate the role of these hypothetical proteins, and valuable data about their potential functionality have been obtained (22–25). Outer membrane proteins play an important role in the virulence mechanisms of pathogens, by acting as adhesins, targets for bactericidal antibodies and receptors for various host molecules (26). In recent years, the reverse vaccinology applied to the genome of *Leptospira* identified putative antigens that were studied using different approaches, which will be discussed along with this review, attempting to find the best way to fight leptospirosis.

Recombinant proteins rLipL21, rLipL32 and rLipL41 were expressed using *E. coli* Bl21 (DE3) hosts, and all proteins were recognized by serum from leptospirosis patients. Hamster lethal challenge with *L interrogans* serovar Lai strain Lai or *L. borgpetersenii* serovar Ballum strain Pishu was performed in animals immunized with each recombinant protein or with co-administered proteins. Animals immunized with co-administration of rLipL21 + rLipL32 + rLipL41 demonstrated the highest survival rate (91.7%). According to Luo et al., these rLipLs could be considered a potential multiple subunit vaccine candidates for use in China (27).

Lsa46 and Lsa77, proteins with OmpA-like domains, have been identified in the genome sequences of *L. interrogans*. The recombinant proteins bind to PLG and plasma fibronectin dose-dependently, and they are immunogenic, eliciting Th1 and Th2 response and capable of reacting with antibodies found in both phases of leptospirosis, onset and convalescent phases (28). When Lsa46 and Lsa77 immunoprotective activity was evaluated in a hamster model of leptospirosis, they induced higher titers of IgG compared to bacterin. In lethal challenge assays, the average survival ratio from two independent experiments was 50% and 44%, respectively for Lsa77 and Lsa46. No statistical difference was found between the immunized groups and PBS control group, when the experiments were analyzed together. In any case, no protein was able to promote renal clearance of *Leptospira*. Nevertheless, when the animals were inoculated with the combination of Lsa46 and Lsa77, protection improved (29). In a recent study, immune protection by Lsa25.6, Lsa16, Lsa14, Lsa19, Lsa24.9, LipL46, rLIC11711 and rLIC13259 adsorbed on Alhydrogel was analyzed. It is known that these proteins are characterized as conserved immunogenic surface proteins and interact with several host components. A high production of IgG was demonstrated in hamsters immunized with all eight antigens, and antibody induction was higher after the first booster, with a partial survival rate of 25 to 42% being observed. In that study, although there was a reduced survival percentage with rLIC11711 and rLIC13259, some bacterial renal clearance was seen, suggesting that both proteins merit further investigation (30).

Leptospiral immunoglobulin-like proteins (Ligs) were first obtained from a DNA genomic library of pathogenic *Leptospira*, *L. interrogans* serovar Pomona, back in 2002 (31). There are three classes of surface-exposed Ligs, namely LigA, LigB and LigC. The Lig sequences are found only in pathogenic *Leptospira* spp. It has been reported that Lig A and Lig B expression is associated with virulence (32). LigA (aa 68-1224) and LigB (aa 68-1191) proteins both confer a high level of protection, but they are unable to promote sterilizing immunity (33). More recently, a recombinant LigB fragment rLigB (131–645), expressed in *E. coli* B21 (DE3), was evaluated as a subunit vaccine and conferred significant protection of 80-100% in a lethal challenge in a hamster model, in seven independent experiments. In surviving vaccinated hamsters, the subunit fragment rLigB (131–645) conferred sterilizing immunity (77.8-100%) as attested by bacterial isolation in renal culture and qPCR. Animals immunized with rLigB (131–645) were able to induce IgG antibody significantly, however when compared with bacterin group these levels were three times lower. When IgG subclasses were evaluated, bacterin group induced an almost exclusive IgG2 response, while the rLigB group produced a mixed IgG1 and IgG2 response. Although vaccinated groups have produced a substantial amount of antibodies, the authors were unable to correlate it to protection (34).

## DNA Vaccines

DNA vaccines are considered a recent approach in vaccine development aiming to achieve a more effective response. DNA vaccination methods provide prolonged expression of the heterologous protein in the animal host and, consequently, long-term exposure to the antigen. It presents a number of advantages over other approaches, such as inexpensive scale-up, improved stability and the possibility to encode more than one gene in a single vaccine (35–37). Moreover, when used in prime-boost strategies to induce a broad and high-level immunity, eliciting both cellular and humoral responses. On the other hand, the drawbacks of DNA vaccines are the lower immunogenicity demonstrated so far in human clinical trials and the development of tolerance in response to long-term exposure to the pathogenic antigen (35, 37, 38). Antigens that have already been or are being studied for the development of a novel *Leptospira* DNA vaccine include proteins highly conserved in pathogenic *Leptospira*, but not found in saprophytic strains, and for this reason have been largely studied as interesting vaccine candidates.

LipL32, also known as hemolysis-associated protein 1 (Hap1), is the most abundant protein in pathogenic *Leptospira* and is absent in saprophytic species. It was extensively tested as a potential antigen against leptospirosis, as it is highly immunogenic in animal models (39–41). LipL32 was the first vaccine against *Leptospira* that was delivered using plasmid DNA (39, 42). That construct, containing the *hap1* gene from *L. interrogans*, in the mammalian expression vector pCDNA3.1, conferred protection against lethal challenge by *L. interrogans* serovar Canicola in gerbils, producing a 60% survival rate, compared to a 35% survival rate in the control group (39).

Another DNA vaccine using the plasmid pTarget/*lipL32* construct was evaluated, comparing it to a recombinant subunit vaccine and a live recombinant BCG vaccine (see below). The three vaccine constructs were able to induce a significant humoral immune response producing antibodies able to recognize the native protein in the intact membrane of *L. interrogans* (43).

The use of a DNA vaccine with the *ompL1* gene was evaluated in Golden Syrian hamsters. The vaccine was found to be well tolerated by the immunized animals and to confer partial protection against heterologous *Leptospira* lethal challenge, and furthermore, it delayed time of death and reduced morbidity and/or the numbers of *Leptospira* in the tissues (44).

An interesting study by Feng et al. (2009) reported humoral and cell immune responses using a prime-boost strategy. In this study, the authors compared the immunization in Balb/c mice with recombinant LipL32 apart or associated with LipL41 and OmpL1 (LipL32-41-OmpL1) by using a DNA and subunit vaccine. Mice immunized with the DNA vaccine containing LipL32-41-OmpL1 or only LipL32 showed an increase in cell-mediated immunity with higher levels of IL-2 and INF-γ than those immunized with homologous proteins, as expected for DNA vaccines. Moreover, the prime boost strategy stimulated more antibodies than a DNA vaccine and showed greater production of cytokines (45).

Another surface-exposed protein of *Leptospira*, OmpL37, displays higher adhesion affinity to elastin tissue compared to other OMPs, and it is believed to play an important role during infection because of its presence in different pathogenic serovars (26, 46, 47). The cited study evaluated the protective immune response of OmpL37-DNA vaccine by using a prime-boost strategies. No induction of protective immune response was found in the group that received the DNA vaccine only. Although a significant IgG response has been observed in prime-boost strategy (48).

LigA and LigB proteins share an almost identical N-terminus sequence referred to as LigBrep and a variable C-terminus portion referred to as LigAni and LigBni (35). One study using both the conserved portion of LigA (LigAcon, amino acids 32 to 626) and the variable portion (LigAvar, amino acids 631 to 1225) from *L. interrogans* serovar Pomona demonstrated that the LigA DNA vaccine significantly increased antibody titers after booster and protected hamsters against lethal challenges, showing 100% survival across three different experiments, compared to the controls with an average survival rate of 62.5% (49). Additionally, it reduced histopathological lesions in vital organs and significantly increased the proliferation of lymphocytes and activation of Th1 and Th2 response after stimulation with the antigens, with no significant differences between LigAcon and LigAvar. In another study, five truncated forms of LigA and LigB from *L. interrogans* serovar Canicola were tested for their protective potential as DNA vaccines. The *ligBrep* construct showed the best results with a 62.5% survival rate in a hamster challenge using *L. interrogans* serovar Copenhageni strain Spool, while the other constructs showed no survival. Additionally, LigBrep induced an IgG response and conferred sterilizing immunity in the surviving hamsters (42). More recently, a recombinant Lig chimera consisting of LigAni and LigBrep was evaluated as a subunit and as a DNA vaccine, and conferred 100% protection in hamsters, but sterilizing immunity was not achieved (50).

LemA is a putative lipoprotein that has similarities with orthologous proteins described in other pathogenic *Leptospira*, therefore being potentially able to stimulate a cross-protective response (51, 52). LemA from *L. interrogans* serovar Copenhageni was tested as subunit, DNA and prime-boost vaccines using a hamster model; this antigen induced different levels of protection, being significant against mortality. An efficacy rate of 62.5% and 87.5% was observed for the DNA and prime-boost strategies, respectively. However, the DNA vaccine did not elicit an antibody response in hamsters, which suggests the involvement of a cellular immune response (51). A more recent study by the same group demonstrated that an rLemA-based DNA vaccine using nanoparticles as carriers was successful in transfecting CHO cells and inducing an IgG response in hamsters. Hamsters were immunized with one of two constructs with different antigen carriers, halloysite clay nanotubes (HCN) and amine-functionalized multi-walled carbon nanotubes (NH2-MWCNTs), and there was 66.7 and 83.3% survival against lethal challenge, respectively (52).

Since the pioneer study of (39) using a DNA construct encoding LipL32 antigen, progress has been made in the field of DNA vaccines against leptospirosis in animal models, proving it to be a practical approach.

## Live Vaccines

The live vaccine approach is based on the use an attenuated auto-replicating microorganism with immunostimulating properties, such as bacillus Calmette-Guerin (BCG) (43, 53) or Adenovirus (54), as a vector for delivering foreign antigens from a pathogen.

Seixas and colleagues used several approaches to screen LipL32 for immune protective activity, i.e., in pTarget as DNA vaccine, in pAE as recombinant protein expressed in *E. coli* and as in live recombinant BCG (rBCG). These constructs were used to immunize mice, and evaluation of their humoral immune response showed the highest antibody titer with rLipL32, while a steady rise in antibodies was detected with LipL32 rBCG (43). Although not evaluated, it is anticipated that rBCG triggers a potent cellular response against heterologous antigens (55). Growth inhibition of *Leptospira* was observed *in vitro* in the presence of anti-LipL32 monoclonal antibodies, stressing the immunogenic capacity of LipL32. Seixas and coworkers further studied the capacity of rBCG expressing LipL32 as antigen against leptospirosis (53). The results showed that animals immunized with different constructs of rBCG/LipL32 promoted seroconversion of total anti-LipL32, with higher titer compared to wild-type BCG, used as control. The protective effect of immunization with rBCG/LipL32 in a leptospirosis hamster model resulted in inconsistent data, varying from 55.88 to 11.76% animal survival, depending on the rBCG construct used, contrasting with 100% survival of animals inoculated with killed whole-leptospires (bacterin). However, histopathological analyses did not show clinical signs of the disease in animals vaccinated with rBCG/LipL32 or bacterin. Furthermore, the authors did not detect bacteria in the lungs and kidneys of these hamsters. The data contrast with the severe pathological signs observed in animals vaccinated with wild-type BCG. These authors highlighted the potential of LipL32 as an antigen against leptospirosis and the importance of rBCG for the delivery of live vaccine. In another study, Oliveira and co-workers (2019) used rBCG combined with a multi-epitope protein approach aimed at finding a system to elicit a protective immune response and prevent renal colonization. They generated two chimeric antigens, one having LipL32, LemA and LigA 11-13 (Q1), and the other LigA 11-13 and LiBrep (Q2). The expression of both chimeric proteins in rBCG under the control of five different mycobacterial promoters was evaluated by Western blotting for protein expression. The immune protection elicited by rBCG expressing Q1 conferred 80 to 100% survival; no bacteria were detected in renal cultures and qPCR data of the cultures were negative. On the other hand, rBCG expressing Q2, resulted in 20 to 60% survivors; the number of bacteria found in renal culture was inconsistent, with sample cultures varying from 2 to 6 out of 10, and qPCR data were positive (56). Another research group extending the above-described results used different recombinant chimera constructs to transform BCG, P1 (*lipL32*), P2 (*ligAni*), P3 (*lemA:ligAni*) and P4 (*lipL32:lemA*) (57). The rBCG expressing these four groups plus wild-type BCG Pasteur control group, was used to vaccinate 10 hamsters, and their results showed that all 4 groups conferred immune protection and prevented renal colonization against challenge with virulent *L. interrogans*. These combined methodologies, rBCG and chimeric multi-epitope proteins, seem to be a very encouraging alternative for the controlling of leptospirosis.

An attenuated transposon mutant of *L. interrogans* serovar Manilae, named M1352, which is defective in lipopolysaccharide biosynthesis, was used as live vaccine to immunize hamsters followed by challenge with the homologous serovar Manilae and other serologically unrelated serovars, e.g., Pomona (58). As a positive control, heat-killed (HK) Manilae bacteria were used. The live M1352 vaccine conferred protection against challenge with homologous and heterologous strains (cross-protection), with higher survival for the live (M1352) compared to killed vaccine. Interesting results were obtained when the authors used immunoblotting from two-dimensional gel electrophoresis aiming to identify proteins from the serum of hamsters inoculated with either killed and or live vaccines. The experiment was conducted with gel electrophoresis of preparations of serovars Manilae and Pomona. Proteins were transferred to a membrane that was probed with antiserum from 5 hamsters of each vaccinated group. Antibodies present in serum of animals immunized with either live or killed vaccine recognized LipL32 and LipL41. Other proteins reacted with both Pomona and Manilae only from hamsters inoculated with the live vaccine. The authors of this study concluded that these antigens are potential vaccine candidates. They are LA3961 (hypothetical protein), Loa22 (OmpA family protein), and LA2372 (general secretory protein G). These data were further extended to four additional leptospiral serovars, aiming to expand cross-protection promoted by M1352 (59). The live attenuated M1352 was able to confer protection against four leptospiral serovars, namely Grippotyphosa, Australis, Canicola and Autumnalis. Strong and significant protection, in terms of animal survival, was observed against the serovars Grippotyphosa (up to 100%), Canicola (62.5–90%) and Australis (75%), with all data compared to the EMJH-vaccinated group. The virulence of serovar Autumnalis strains was inconsistent, but protection was observed with two strains tested, although less marked than for the other serovars tested. The live vaccine elicited no significant protection against renal colonization not even with homologous bacterins, corroborating the previous findings with M1352, which promoted significant but incomplete protection against renal colonization for a homologous challenge, and insignificant protection against the heterologous serovar Pomona (58). The data obtained thus far with different antigens are inconsistent in promoting animal survival or conferring sterilizing immunity, an important point to prevent transmission between animal hosts. Hence, preventing renal colonization remains a great challenge in the development of vaccines against leptospirosis.

Another work using live attenuated leptospiral vaccine characterized a motility-deficient mutant lacking the expression of a flagellar coil protein, FcpA. Wunder and colleagues showed that these *L. interrogans* L1-130 mutants lose their capacity to generate translational motility and to penetrate mucous membranes, resulting in loss of kidney colonization and hamster lethality. These investigators evaluated whether the fcpA- motility-deficient mutant had the capacity to act as a live attenuated vaccine. Despite having an attenuation phenotype, the mutant strain inoculated in hamsters or in mice produced a transient bacteremia, which was sufficient to promote strong anti-*Leptospira* antibodies (60). Similar to the work by Srikram et al. (58) with attenuated transposon mutant of *L. interrogans* serovar Manilae, these are protein antibodies and not agglutinating antibodies. These antibodies are correlates of fcpA-attenuated vaccine and are responsible for mediating cross-protective immunity. Using proteome from the sera of hamsters or mice inoculated with fcpA-mutant-attenuated vaccine, identified 154 proteins, which represent important targets that can be used for the development of vaccines against and/or diagnostics for leptospirosis.

Vernel-Pauillac and colleagues studied over 6 months the antibody immune response of mice infected with three pathogenic serovars, Manilae, Copenhageni and Icterohaemorrhagiae, attenuated mutants or heat-killed bacteria. Anti-*Leptospira* IgGs including, IgA, IgM, IgG and 4 subclasses were assessed, and their cross-reactivity among serovars evaluated. The level of protection of these immunized animals was evaluated after *L. interrogans* challenge. Pre-inoculated mice challenged with high doses of homologous bacteria showed response boosting of all antibodies and were protected against leptospirosis. Interestingly, the pre-inoculation of mice with the attenuated M895 Manilae LPS mutant or heat-killed bacterin prevented renal colonization 2 months after the challenge. The authors also showed that in mice the type of immune response post-*L. interrogans* challenge was dependent on the serovar and virulence of the strains used (61). Although there is criticism of the animal model since mice are resistant to leptospirosis, in contrast to humans which are susceptible to developing a life-threatening disease, the mouse model would be interesting for a better understanding of host immunity against *Leptospira* and to study mechanisms associated with long-term protection of the disease and the dependence on serovar. Albeit there is a lack of reagents directed to hamster, it will be important to extend these studies to this leptospirosis animal model.

## Multi-Epitope Vaccines

One of the strategies studied includes the multi-epitope vaccines. The rational of this approach is based on epitopes of proteins previously studied, presenting either partial or full immune protective activity and eliciting partial or no sterilizing protection in the hamster model of leptospirosis.

In 2008, Lin et al. reported the construction of a recombinant multi-epitope protein of *Leptospira* (r-LMP) that could detect anti-leptospirosis antibodies in patient sera. Using phage display and immunity reaction, immunodominant epitopes of the outer membrane proteins OmpL1, LipL21 and LipL32 were selected. Based on the generated sequences, five major immunodominant epitopes were identified and used to construct a synthetic gene, called recombinant *lmp*. The gene was cloned and the protein expressed using *Escherichia coli* as the host expression system. The purified recombinant multi-epitope protein was recognized by antigens in leptospirosis serum samples, even when the microagglutination test (MAT) was negative; that is, the serum was not yet converted (62). In a subsequent work, this research group using this recombinant chimeric multi-epitope vaccine, consisting of four repeats of six T- and B-cell combined epitopes of the same proteins, OmpL1, LipL32, and LipL21, named r4R, tested the capacity of this protein to elicit a protective immune response in guinea pigs. The data presented was very promising, showing increased survival and reduced renal colonization, compared to the control PBS group, against the lethal challenge with virulent *Leptospira* (63).

In another work, the designed chimeric protein was based on the amino acid sequences of previously studied outer membrane proteins, namely LigA, Mce, Lsa45, OmpL1 and LipL41. The amino acid sequences chosen were: 852 to 1107 of LigA, 131 to 207 of Mce, 190 to 250 of Lsa45, 153 to 221 of OmpL1 and 213 to 276 of LipL41. The selected amino acid regions were based on experimental work using an animal model (64–68). The amino acids of different proteins were linked with the flexible peptide (GGGGSGGGGSGGGGS), allowing independent folding of each region. This multi-epitope sequence was cloned and the chimeric protein expressed in *E. coli*. The multi-epitope chimeric protein (rChi) was able to confer partial immune protection in leptospirosis hamster challenge when administered with either Alhydrogel or *Bordetella pertussis* monophosphoryl lipid A (MPLA) adjuvants. Noteworthy was the high reactivity of the chimeric protein observed with serum samples of experimentally infected hamster when compared to the normal hamster serum samples. High reactivity was observed when the rChi protein was tested with human leptospirosis serum samples in the convalescent phase of disease (MAT-positive), while only a few samples were reactive at the onset of the disease (MAT-negative) (69). More studies are needed with the rChi protein to understand the type of immune response that is promoted in hamsters, as well as, if leptospiral renal colonization could be prevented.

The work reported by Validi and colleagues used bioinformatics to design the multi-epitope vaccine. The predicted T cell and IFN gamma epitopes of the antigens LipL32, LigA, *Leptospira* antigen of 42 kDa (LAg42), hemolysin SphH and heat-shock protein 58 (HSP58) (54, 64, 70–74) were linked using EAAAK, GPGPG, AAY and HEYGAEALERAG. To improve the immune response, they incorporated the construct heparin-binding hemagglutinin (HBHA), as an adjuvant. A multi-epitope vaccine construct of 490 amino acids was generated and several physico-chemical properties among others were evaluated by using immunoinformatics analyses, as described in the literature (75, 76) and the following webservers, http://www.imtech.res.in/raghava/propred/, http://tools.iedb.org/. These analyses strongly suggest that this vaccine is a competent antigen for immune response against leptospirosis, the authors also discuss the use of this multi-epitope vaccine for prophylactic or therapeutic uses. Although an interesting approach, experimental studies are needed before this *in silico* methodology can be validated, including the immune protective assay in animal models.

Da Cunha and colleagues evaluated the immunoprotective activity of a chimeric fusion of LigA non-identical fragment (LigAni) and LigB repetitive fragment (LigBrep), generating a final construct named Lig chimero (LC). Among other formulations evaluated, hamsters immunized with LC elicited a high humoral response and the survival rate in a lethal challenge with *L. interrogans* strain Fiocruz L1-130 was the same as that observed with bacterin as vaccine (100%). When rLC was adsorbed to Montanide, a higher titer of IgG2/3 than IgG1 was detected, indicating a polarized Th1 response. In contrast, administration of rLC with alum demonstrated a mixed Th1 and Th2 response and elicited the same protection as the first preparation. Although both preparations resulted in complete survival of hamsters, they were not able to produce sterilizing immunity (50).

As an alternative to this approach, Garba and collaborators (2018) described an *in silico* design of multi-epitope DNA vaccine composed of the immunogenic epitopes of LipL32 and LipL41. Five antigenic epitopes, 2 of LipL32 and 3 of LipL41 were predicted by bioinformatics and linked together with the help of a GGGGS spacer between them. To stimulate the immune capacity of the multi-epitope chimeric gene construct, a CpG motif was added at both the 5′ and 3′ end of the gene. This multi-epitope DNA vaccine, administered in hamsters as a plasmid DNA vaccine, showed partial protection against lethal leptospires, induced both agglutinating and neutralizing antibodies, as confirmed by MAT and *in vitro* growth inhibition assays, and was capable of reducing renal colonization. Renal histopathological lesions were further analyzed and mild to moderate pathologies were found, in contrast to severe lesions observed in the control group (77). The immunogenic multi-epitope DNA vaccines represent an interesting approach, as epitopes from several immunogenic leptospiral proteins could be tested to develop a novel vaccine against leptospirosis.

## Use of Toll-Like and Nod-Like Receptor Agonists as a Novel Vaccine Strategy

Toll-like receptors (TLRs) and nod-like receptors (NLRs) are pattern recognition receptors (PRRs), which recognize pathogen-associated molecular patterns (PAMPs) leading to activation of innate immunity, and they can promote the activation of antigen-specific adaptive immunity (78, 79). The idea of using TLR and NLR agonists as vaccine targets to prevent leptospirosis emerged from studies that show the differential involvement of leptospires between hosts with the TLR2, TLR4, TLR5 and NOD receptors, which could explain the cases of susceptibility and resistance to the disease (80–84). First, it was observed that the co-injection of a TLR2 agonist (Pam3CSK) with virulent leptospires increased survival rate and reduced the bacterial burden in the kidneys, liver and lungs of hamsters when compared to control animals. The injection of Pam3CSK4 6 h after infection also improved survival, but it was inferior when compared to co-injection, suggesting that an early activation of TLR2 could be necessary for protection (85). Along the same line, LPS from *E. coli*, a very well-characterized TLR4 agonist, when administered 24 h before infection followed by 3 days post-infection, produced 100% survival. Untreated hamsters died in the first days after infection, while the administration of a single dose of LPS 24 h post-infection generated about 50% survival, reinforcing the notion that an early inflammatory response is important to control leptospirosis (86). An interesting observation in those studies was that while Pam3CSK4 showed a tendency to reduce the bacterial burden in organs; this was not observed after using LPS. Moreover, histopathological analysis revealed a more exacerbated inflammatory profile for LPS treatment than for Pam3CSK4.

In another study performed with β-glucans, similar survival data were obtained. This glucose polymer found in the wall of yeast cells is known to activate innate immunity through interaction or regulation of several receptors, including TLR receptors. All hamsters that received a high concentration of β-glucan one day before and after infection survived the challenge with virulent leptospires and showed a decrease in bacterial burden and milder histopathological signs (87, 88). Both animals treated with β-glucan and LPS showed an increased expression of TLR2 in the kidneys 2 days post-infection, strongly suggesting that early TLR2 activation could be responsible for the survival observed. The response profile obtained in hamsters treated with β-glucans was similar to infection developed by mice, a resistant animal model of leptospirosis. These resistant animals begin to express TLR2 from 6 h post-infection reaching a peak at 24 h, while hamsters show late expression. TLR4 expression is not induced in either animal model at early infection (85). However, animals that received LPS showed substantial TLR4 expression 2 days after infection. Perhaps, it could explain why animals that received LPS were not able to eliminate leptospires and showed an exacerbated inflammatory response.

A new approach of prophylactic strategy has been highlighted after an observation that *Lactobacillus plantarum* treatment of C3H/Hej mice was able to minimize leptospirosis severity after sub-lethal challenge (89). *Lactobacillus plantarum* known to have immunomodulatory and probiotic properties (90, 91), when administered orally in a total of 30 doses before infection, regulates the inflammatory response and reduces the renal damage caused by the disease. Despite this treatment showing a tendency to reduce the number of *Leptospira* in blood and urine, did not prevent renal colonization, possibly because this response was not specific as observed in a secondary immune response. In many studies of the gastrointestinal tract, the administration of probiotic bacteria promotes a decrease in inflammatory response *via* strain-specific interactions with TLRs and NLRs (92–95). Nevertheless, the idea of using PRR agonists as a novel vaccine strategy to fight leptospirosis seems to be quite valuable and deserves to be further investigated.

In the case of a lethal mouse pneumovirus infection, it has been shown that *Lactobacillus plantarum* administered directly to the respiratory tract is able to protect mice against infection by mediating *Lactobacillus plantarum* engagement with TLR2 and NOD2 receptors (96–98). The involvement of these two ligands in the modulation of the virus-induced inflammatory process was confirmed later by using a bi-functional NOD2-TLR2 ligand, named CL429 (98). Interestingly, the administration of CL429 ligand in mice some days before infection with *L. interrogan*s MFLum1 strain promoted reduction of leptospirosis signs similar to those observed with the *Lactobacillus plantarum* administration. The response obtained in CL429-treated mice was associated with an innate immune memory response, since *ex vivo* data showed an increase in pro-inflammatory mediators and nitric oxide production by peritoneal macrophages, bone marrow-derived macrophages and splenic cells after stimulation with leptospires. The bactericidal activity of cells from CL429-treated mice was also confirmed, as well as the cytokine production independent of adaptive immune cells. Interestingly, although the profile of innate immune memory cells was sustained for 3 months, *in vivo* data show that animals were able to control the kidney load only in the first 15 days post-infection (99). As these experiments were performed in mice, it is still unclear if the same response profile will be observed in a hamster model and if the animals could survive a lethal challenge. Nevertheless, the idea of using PRR agonists as a novel vaccine strategy to fight leptospirosis seems to be quite promising and deserves to be investigated in depth.

## Future Perspectives

The post-genomics era enabled the development of the so-called “second-generation vaccines”. To overcome the drawbacks of leptospirosis bacterin vaccines, the elucidation of the *Leptospira* genome (17, 100) was a turning point for the new generation vaccines. In this regard, the study of recombinant subunit vaccines appeared to be a very stimulating and promising prospect (101). Despite all the remarkable advances achieved within these studies, at the moment they have been shown to elicit only partial protection against *Leptospira* challenge (102, 103), and the majority of them fail to prevent renal colonization. The future of subunit vaccines against leptospirosis should focus not only on the discovery of new antigens but on the use of multicomponent antigens, and also on the use of adjuvants that could direct to humoral and cellular immune response pathways.

The biotechnological advances in the vaccine field, the emerging development of nucleic acid vaccines – called the “third-generation DNA and RNA vaccines”– was made possible owing to the elucidation of several genomes (104, 105). These third-generation vaccine approaches aim to vaccinate a patient with genetic material that encodes the target antigen. As discussed earlier, a considerable number of DNA vaccines against leptospirosis have been tested in recent years, yet mRNA vaccines against leptospirosis have not been tested.

mRNA vaccines have been considered as a promising new era in vaccinology (105). They have the advantages of simplicity and convenience that are shared with DNA vaccines, but they lack the disadvantages of the difficulty of controlling prolonged expression through integration in the host genome, thereby inducing immunological tolerance against target genes (38). Moreover, exogenous single-stranded mRNA is an innate immune stimulant acting as a strong PAMP recognized by pattern receptors on the cell surface and elicit robust B and T cell responses (106).

Until recently, mRNA vaccines have been studied in cancer treatment (107) and against some viruses such as chikungunya (108) and zika (109). During 2020, with the proof of efficacy of mRNA vaccines against SARS-CoV-2 Covid-19 pandemic produced by BioNTech and Pfizer (110) and Moderna (111), the future for developing this kind of vaccine has become more than a promise but a reality. Therefore, the use of mRNA vaccines targeting multicomponent antigens and the use of adjuvants may be a very attractive approach to achieve an efficient vaccine against *Leptospira* infection.

## Author Contributions

All authors participated in the literature revision, discussion and preparation of manuscript.

## Funding

The following Brazilian agencies: AN, AL, and GB have FAPESP grants (2019/17488-2, 2018/05823-9 and 2018/10384-4), AN has CNPq grant (301229/2017-1) and Fundação Butantan, financially supported this work; AT has FAPESP fellowship (2016/11541-0); BOPA has a fellowship from CNPq, DKD and MPO have fellowships from CAPES. The funders had no role in study design, analysis, decision to publish, or preparation of the manuscript.

## Conflict of Interest

The authors declare that the research was conducted in the absence of any commercial or financial relationships that could be construed as a potential conflict of interest.

## Publisher’s Note

All claims expressed in this article are solely those of the authors and do not necessarily represent those of their affiliated organizations, or those of the publisher, the editors and the reviewers. Any product that may be evaluated in this article, or claim that may be made by its manufacturer, is not guaranteed or endorsed by the publisher.

## References

[1] Adler B de la Peña Moctezuma A. *Leptospira* and Leptospirosis. Vet Microbiol (2010) 140(3-4):287–96. doi: 10.1016/j.vetmic.2009.03.012 19345023

[2] Gouveia EL Metcalfe J de Carvalho ALF Aires TSF C V-BJ Queirroz A. Leptospirosis-Associated Severe Pulmonary Hemorrhagic Syndrome, Salvador, Brazil. Emerg Infect Dis (2008) 14(3):505–8. doi: 10.3201/eid1403.071064 PMC2570821 18325275

[3] Bharti AR Nally JE Ricaldi JN Matthias MA Diaz MM Lovett MA. Leptospirosis: A Zoonotic Disease of Global Importance. Lancet Infect Dis (2003) 3(12):757–71. doi: 10.1016/S1473-3099(03)00830-2 14652202

[4] Faine S Adler B Bolin C Perolat P. Leptospira and Leptospirosis. 2nd Edition. Melbourne: Medisci Press (1999).

[5] Vincent AT Schiettekatte O Goarant C Neela VK Bernet E Thibeaux R. Revisiting the Taxonomy and Evolution of Pathogenicity of the Genus *Leptospira* Through the Prism of Genomics. PLoS Negl Trop Dis (2019) 13(5):e0007270. doi: 10.1371/journal.pntd.0007270 31120895 PMC6532842

[6] Haake DA Levett PN. Leptospirosis in Humans. Curr Top Microbiol Immunol (2015) 387:65–97. doi: 10.1007/978-3-662-45059-8_5 25388133 PMC4442676

[7] Xu Y Ye Q. Human Leptospirosis Vaccines in China. Hum Vaccin Immunother (2018) 14(4):984–93. doi: 10.1080/21645515.2017.1405884 PMC5893195 29148958

[8] Adler B. Vaccines Against Leptospirosis. Curr Top Microbiol Immunol (2015) 387:251–72. doi: 10.1007/978-3-662-45059-8_10 25388138

[9] Laurichesse H Gourdon F Smits HL Abdoe TH Estavoyer JM Rebika H. Safety and Immunogenicity of Subcutaneous or Intramuscular Administration of a Monovalent Inactivated Vaccine Against *Leptospira Interrogans* Serogroup Icterohaemorrhagiae in Healthy Volunteers. Clin Microbiol Infect (2007) 13(4):395–403. doi: 10.1111/j.1469-0691.2007.01662.x 17359323

[10] Martinez R Pérez A Quiñones C Cruz R Alvarez A Armesto M. Efficacy and Safety of a Vaccine Against Human Leptospirosis in Cuba. Rev Panam Salud Publica (2004) 14(4):249–55. doi: 10.1590/s1020-49892004000400005 15193180

[11] Rappuoli R. Reverse Vaccinology. Curr Opin Microbiol (2000) 3:445–50. doi: 10.1016/S1369-5274(00)00119-3 11050440

[12] Adu-Bobie J Capecchi B Serruto D Rappuoli R Pizza M. Two Years Into Reverse Vaccinology. Vaccine (2003) 21:605–10. doi: 10.1016/S0264-410X(02)00566-2 12531326

[13] Rappuoli R Black S Bloom DE. Vaccines and Global Health: In Search of a Sustainable Model for Vaccine Development and Delivery. Sci Transl Med (2019) 11:eaaw2888. doi: 10.1126/scitranslmed.aaw2888 31217336

[14] Sette A Rappuoli R. Reverse Vaccinology: Developing Vaccines in the Era of Genomics. Immunity (2010) 33(4):530–41. doi: 10.1016/j.immuni.2010.09.017 PMC3320742 21029963

[15] Serruto D Bottomley MJ Ram S Giuliani MM Rappuoli R. The New Multicomponent Vaccine Against Meningococcal Serogroup B, 4cmenb: Immunological, Functional and Structural Characterization of the Antigens. Vaccine (2012) 30(Suppl 2):B87–97. doi: 10.1016/j.vaccine.2012.01.033 PMC3360877 22607904

[16] Rappuoli R Pizza M Masignani V Vadivelu K. Meningococcal B Vaccine (4cmenb): The Journey From Research to Real World Experience. Expert Rev Vaccines (2018) 17(12):1111–21. doi: 10.1080/14760584.2018.1547637 30457407

[17] Ren SX Fu G Jiang XG Zeng R Miao YG Xu H. Unique Physiological and Pathogenic Features of *Leptospira Interrogans* Revealed by Whole-Genome Sequencing. Nature (2003) 422(6934):888–93. doi: 10.1038/nature01597 12712204

[18] Nascimento ALTO Verjovski-Almeida S Van Sluys MA Monteiro-Vitorello CB Camargo LEA Digiampietri LA. Genome Features of *Leptospira Interrogans* Serovar Copenhageni. Braz J Med Biol Res (2004) 37:459–78. doi: 10.1590/S0100-879X2004000400003 PMC2666282 15064809

[19] Nascimento ALTO Ko AI Martins EAL Monteiro-Vitorello CB Ho PL Haake DA. Comparative Genomics of Two *Leptospira Interrogans* Serovars Reveals Novel Insights Into Physiology and Pathogenesis. J Bacteriol (2004) 186(7):2164–72. doi: 10.1128/JB.186.7.2164-2172.2004 PMC374407 15028702

[20] Bulach DM Zuerner RL Wilson P Seemann T McGrath A Cullen PA. Genome Reduction in *Leptospira Borgpetersenii* Reflects Limited Transmission Potential. PNAS (2006) 103(39):14560–5. doi: 10.1073/pnas.0603979103 PMC1599999 16973745

[21] Picardeau M Bulach DM Bouchier C Zuerner RL Zidane N Wilson PJ. Genome Sequence of the Saprophyte *Leptospira Biflexa* Provides Insights Into the Evolution of *Leptospira* and the Pathogenesis of Leptospirosis. PLoS One (2008) 3(2):e1607. doi: 10.1371/journal.pone.0001607 18270594 PMC2229662

[22] Fernandes LG Siqueira GH Teixeira ARF Silva LP Figueredo JM Cosate MR. *Leptospira* Spp.: Novel Insights Into Host-Pathogen Interactions. Vet Immunol Immunopathol (2016) 176:50–7. doi: 10.1016/j.vetimm.2015.12.004 26727033

[23] Cavenague MF Teixeira AF Filho AS Souza GO Vasconcellos SA Heinemann MB. Characterization of a Novel Protein of *Leptospira Interrogans* Exhibiting Plasminogen, Vitronectin and Complement Binding Properties. Int J Med Microbiol (2019) 309(2):116–29. doi: 10.1016/j.ijmm.2018.12.005 30638770

[24] Kochi LT Fernandes LGV Souza GO Vasconcellos SA Heinemann MB Romero EC. The Interaction of Two Novel Putative Proteins of *Leptospira Interrogans* With E-Cadherin, Plasminogen and Complement Components With Potential Role in Bacterial Infection. Virulence (2019) 10(1):734–53. doi: 10.1080/21505594.2019.1650613 PMC6735628 31422744

[25] Passalia FJ Carvalho E Heinemann MB Vieira ML Nascimento A. The *Leptospira Interrogans* LIC10774 Is a Multifunctional Surface Protein That Binds Calcium and Interacts With Host Components. Microbiol Res (2020) 235:13. doi: 10.1016/j.micres.2020.126470 32247916

[26] Pinne M Haake DA. A Comprehensive Approach to Identification of Surface-Exposed, Outer Membrane-Spanning Proteins of *Leptospira Interrogans* . PLoS One (2009) 4(6):e6071. doi: 10.1371/journal.pone.0006071 19562037 PMC2698987

[27] Luo D Xue F Ojcius DM Zhao J Mao Y Li L. Protein Typing of Major Outer Membrane Lipoproteins From Chinese Pathogenic *Leptospira* Spp. And Characterization of Their Immunogenicity. Vaccine (2009) 28(1):243–55. doi: 10.1016/j.vaccine.2009.09.089 19796723

[28] Teixeira AF de Morais ZM Kirchgatter K Romero EC Vasconcellos SA Nascimento A. Features of Two New Proteins With OmpA-Like Domains Identified in the Genome Sequences of *Leptospira Interrogans* . PLoS One (2015) 10(4):25. doi: 10.1371/journal.pone.0122762 PMC4388678 25849456

[29] Teixeira AF Fernandes LGV Souza Filho A Souza GO Vasconcellos SA Heinemann MB. Evaluation of Lsa46 and Lsa77 Leptospiral Proteins for Their Immunoprotective Activities in Hamster Model of Leptospirosis. BioMed Res Int (2018) 2018:1813745. doi: 10.1155/2018/1813745 29984227 PMC6015724

[30] Teixeira AF Cavenague MF Kochi LT Fernandes LG Souza GO de Souza Filho AF. Immunoprotective Activity Induced by Leptospiral Outer Membrane Proteins in Hamster Model of Acute Leptospirosis. Front Immunol (2020) 11:568694. doi: 10.3389/fimmu.2020.568694 33193344 PMC7662565

[31] Palaniappan RU Chang YF Jusuf SS Artiushin S Timoney JF McDonough SP. Cloning and Molecular Characterization of an Immunogenic LigA Protein of *Leptospira Interrogans* . Infect Immun (2002) 70(11):5924–30. doi: 10.1128/IAI.70.11.5924-5930.2002 PMC130282 12379666

[32] Matsunaga J Barocchi MA Croda J Young TA Sanchez Y Siqueira I. Pathogenic *Leptospira* Species Express Surface-Exposed Proteins Belonging to the Bacterial Immunoglobulin Superfamily. Mol Microbiol (2003) 49(4):929–45. doi: 10.1046/j.1365-2958.2003.03619.x PMC1237129 12890019

[33] Koizumi N Watanabe H. Leptospiral Immunoglobulin-Like Proteins Elicit Protective Immunity. Vaccine (2004) 22(11-12):1545–52. doi: 10.1016/j.vaccine.2003.10.007 15063580

[34] Conrad NL Cruz McBride FW Souza JD Silveira MM Felix S Mendonca KS. LigB Subunit Vaccine Confers Sterile Immunity Against Challenge in the Hamster Model of Leptospirosis. PLoS Negl Trop Dis (2017) 11(3):e0005441. doi: 10.1371/journal.pntd.0005441 28301479 PMC5370146

[35] Silveira MM Oliveira TL Schuch RA McBride AJA Dellagostin OA Hartwig DD. DNA Vaccines Against Leptospirosis: A Literature Review. Vaccine (2017) 35(42):5559–67. doi: 10.1016/j.vaccine.2017.08.067 28882437

[36] Xu Y Yuen PW Lam JK. Intranasal DNA Vaccine for Protection Against Respiratory Infectious Diseases: The Delivery Perspectives. Pharmaceutics (2014) 6(3):378–415. doi: 10.3390/pharmaceutics6030378 25014738 PMC4190526

[37] Bashiru G Bahaman AR. Advances & Challenges in Leptospiral Vaccine Development. Indian J Med Res (2018) 147(1):15–22. doi: 10.4103/ijmr.IJMR_1022_16 29749356 PMC5967211

[38] Coban C Kobiyama K Jounai N Tozuka M Ishii KJ. DNA Vaccines: A Simple DNA Sensing Matter? Hum Vaccin Immunother (2013) 9(10):2216–21. doi: 10.4161/hv.25893 PMC3906407 23912600

[39] Branger C Chatrenet B Gauvrit A Aviat F Aubert A Bach JM. Protection Against *Leptospira Interrogans* Sensu Lato Challenge by DNA Immunization With the Gene Encoding Hemolysin-Associated Protein 1. Infect Immun (2005) 73(7):4062–9. doi: 10.1128/IAI.73.7.4062-4069.2005 PMC1168576 15972494

[40] Zhang XY Yu Y He P Zhang YX Hu BY Yang Y. Expression and Comparative Analysis of Genes Encoding Outer Membrane Proteins LipL21, LipL32 and OmpL1 in Epidemic Leptospires. Acta Biochim Biophys Sin (Shanghai) (2005) 37(10):649–56. doi: 10.1111/j.1745-7270.2005.00094.x 16215631

[41] Guerreiro H Croda J Flannery B Mazel M Matsunaga J Galvao Reis M. Leptospiral Proteins Recognized During the Humoral Immune Response to Leptospirosis in Humans. Infect Immun (2001) 69(8):4958–68. doi: 10.1128/IAI.69.8.4958-4968.2001 PMC98588 11447174

[42] Forster KM Hartwig DD Seixas FK Bacelo KL Amaral M Hartleben CP. A Conserved Region of Leptospiral Immunoglobulin-Like A and B Proteins as a DNA Vaccine Elicits a Prophylactic Immune Response Against Leptospirosis. Clin Vaccine Immunol (2013) 20(5):725–31. doi: 10.1128/CVI.00601-12 PMC3647749 23486420

[43] Seixas FK Fernandes CH Hartwig DD Conceição FR Dellagostin OA. Evaluation of Different Ways of Presenting LipL32 to the Immune System With the Aim of Developing a Recombinant Vaccine Against Leptospirosis. Can J Microbiolol (2007) 53(4):472–9. doi: 10.1139/w06-138 17612601

[44] Maneewatch S Tapchaisri P Sakolvaree Y Klaysing B Tongtawe P Chaisri U. OmpL1 DNA Vaccine Cross-Protects Against Heterologous *Leptospira* Spp. Challenge. Asian Pac J Allergy Immunol (2007) 25(1):75–82. 17891923

[45] Feng CY Li QT Zhang XY Dong K Hu BY Guo XK. Immune Strategies Using Single-Component LipL32 and Multi-Component Recombinant LipL32-41-OmpL1 Vaccines Against *Leptospira* . Braz J Med Biol Res (2009) 42(9):796–803. doi: 10.1590/S0100-879X2009005000013 19649391

[46] Pinne M Choy HA Haake DA. The OmpL37 Surface-Exposed Protein Is Expressed by Pathogenic *Leptospira* During Infection and Binds Skin and Vascular Elastin. PLoS Negl Trop Dis (2010) 4(9):e815. doi: 10.1371/journal.pntd.0000815 20844573 PMC2935396

[47] Matsui M Soupé ME Becam J Goarant C. Differential *In Vivo* Gene Expression of Major *Leptospira* Proteins in Resistant or Susceptible Animal Models. Appl Environ Microbiol (2012) 78(17):6372–6. doi: 10.1128/AEM.00911-12 PMC3416592 22729538

[48] Oliveira TL Grassmann AA Schuch RA Seixas Neto AC Mendonça M Hartwig DD. Evaluation of the *Leptospira Interrogans* Outer Membrane Protein OmpL37 as a Vaccine Candidate. PLoS One (2015) 10(11):e0142821. doi: 10.1371/journal.pone.0142821 26588685 PMC4654524

[49] Faisal SM Yan W Chen CS Palaniappan RU McDonough SP Chang YF. Evaluation of Protective Immunity of Leptospira Immunoglobulin Like Protein A (LigA) DNA Vaccine Against Challenge in Hamsters. Vaccine (2008) 26(2):277–87. doi: 10.1016/j.vaccine.2007.10.029 18055070

[50] da Cunha CEP Bettin EB Bakry A Neto A Amaral MG Dellagostin OA. Evaluation of Different Strategies to Promote a Protective Immune Response Against Leptospirosis Using a Recombinant LigA and LigB Chimera. Vaccine (2019) 37(13):1844–52. doi: 10.1016/j.vaccine.2019.02.010 30826147

[51] Hartwig DD Forster KM Oliveira TL Amaral M McBride AJ Dellagostin OA. A Prime-Boost Strategy Using the Novel Vaccine Candidate, LemA, Protects Hamsters Against Leptospirosis. Clin Vaccine Immunol (2013) 20(5):747–52. doi: 10.1128/CVI.00034-13 PMC3647757 23515012

[52] Oliveira TL Bacelo KL Forster KM Ilha V Rodrigues OE Hartwig DD. DNA Nanovaccines Prepared Using LemA Antigen Protect Golden Syrian Hamsters Against *Leptospira* Lethal Infection. Mem Inst Oswaldo Cruz (2020) 115:e190396. doi: 10.1590/0074-02760190396 32321154 PMC7164400

[53] Seixas FK da Silva EF Hartwig DD Cerqueira GM Amaral M Fagundes MQ. Recombinant *Mycobacterium Bovis* BCG Expressing the LipL32 Antigen of *Leptospira Interrogans* Protects Hamsters From Challenge. Vaccine (2007) 26(1):88–95. doi: 10.1016/j.vaccine.2007.10.052 18063449

[54] Branger C Sonrier C Chatrenet B Klonjkowski B Ruvoen-Clouet N Aubert A. Identification of the Hemolysis-Associated Protein 1 as a Cross-Protective Immunogen of *Leptospira Interrogans* by Adenovirus-Mediated Vaccination. Infect Immun (2001) 69(11):6831–8. doi: 10.1128/IAI.69.11.6831-6838.2001 PMC100061 11598056

[55] Teo SS Shingadia D. BCG Vaccine. In: Pollard AJ, editor. Adv Exp Med Biol. Boston, MA: Springer (2005). p. 117–34. 568. 2005/08/19. 10.1007/0-387-25342-4_8 16107069

[56] Oliveira TL Rizzi C da Cunha CEP Dorneles J Seixas Neto ACP Amaral MG. Recombinant BCG Strains Expressing Chimeric Proteins Derived From *Leptospira* Protect Hamsters Against Leptospirosis. Vaccine (2019) 37(6):776–82. doi: 10.1016/j.vaccine.2018.12.050 30630695

[57] Dorneles J Madruga AB Seixas Neto ACP Rizzi C Bettin EB Hecktheuer AS. Protection Against Leptospirosis Conferred by *Mycobacterium Bovis* BCG Expressing Antigens From *Leptospira Interrogans* . Vaccine (2020) 38(51):8136–44. doi: 10.1016/j.vaccine.2020.10.086 33176938

[58] Srikram A Zhang K Bartpho T Lo M Hoke DE Sermswan RW. Cross-Protective Immunity Against Leptospirosis Elicited by a Live, Attenuated Lipopolysaccharide Mutant. J Infect Dis (2011) 203(6):870–9. doi: 10.1093/infdis/jiq127 PMC3071135 21220775

[59] Murray GL Simawaranon T Kaewraemruaen C Adler B Sermswan RW. Heterologous Protection Elicited by a Live, Attenuated, *Leptospira* Vaccine. Vet Microbiol (2018) 223:47–50. doi: 10.1016/j.vetmic.2018.07.018 30173751

[60] Wunder EA Adhikarla H Hamond C Owers Bonner KA Liang L Rodrigues CB. A Live Attenuated-Vaccine Model Confers Cross-Protective Immunity Against Different Species of the *Leptospira* Genus. Elife (2021) 10:1–20. doi: 10.7554/eLife.64166 PMC7837694 33496263

[61] Vernel-Pauillac F Murray GL Adler B Boneca IG Werts C. Anti-*Leptospira* Immunoglobulin Profiling in Mice Reveals Strain Specific IgG and Persistent IgM Responses Associated With Virulence and Renal Colonization. PLoS Negl Trop Dis (2021) 15(3):e0008970. doi: 10.1371/journal.pntd.0008970 33705392 PMC8007020

[62] Lin X Chen Y Yan J. Recombinant Multiepitope Protein for Diagnosis of Leptospirosis. Clin Vaccine Immunol (2008) 15(11):1711–4. doi: 10.1128/CVI.00189-08 PMC2583528 18827193

[63] Lin X Xiao G Luo D Kong L Chen X Sun D. Chimeric Epitope Vaccine Against *Leptospira Interrogans* Infection and Induced Specific Immunity in Guinea Pigs. BMC Microbiol (2016) 16(1):241. doi: 10.1186/s12866-016-0852-y 27737644 PMC5064800

[64] Silva EF Medeiros MA McBride AJ Matsunaga J Esteves GS Ramos JG. The Terminal Portion of Leptospiral Immunoglobulin-Like Protein LigA Confers Protective Immunity Against Lethal Infection in the Hamster Model of Leptospirosis. Vaccine (2007) 25(33):6277–86. doi: 10.1016/j.vaccine.2007.05.053 PMC1994161 17629368

[65] Coutinho ML Choy HA Kelley MM Matsunaga J Babbitt JT Lewis MS. A LigA Three-Domain Region Protects Hamsters From Lethal Infection by *Leptospira Interrogans* . PLoS Negl Trop Dis (2011) 5(12):e1422. doi: 10.1371/journal.pntd.0001422 22180800 PMC3236721

[66] Haake DA Mazel MK Mccoy AM Milward F Chao G Matsunaga J. Leptospiral Outer Membrane Proteins OmpL1 and LipL41 Exhibit Synergistic Immunoprotection. Infect Immun (1999) 67:6572–82. doi: 10.1128/IAI.67.12.6572-6582.1999 PMC97069 10569777

[67] Zhang Y Lou XL Yang HL Guo XK Zhang XY He L. Establishment of a Leptospirosis Model in Guinea Pigs Using an Epicutaneous Inoculations Route. BMC Infect Dis (2012) 12(20):1–10. doi: 10.1186/1471-2334-12-20 22273178 PMC3329641

[68] Fernandes LG Vieira ML Alves IJ de Morais ZM Vasconcellos SA Romero EC. Functional and Immunological Evaluation of Two Novel Proteins of *Leptospira* Spp. Microbiology (2014) 160(Pt 1):149–64. doi: 10.1099/mic.0.072074-0 24162609

[69] Fernandes LGV Teixeira AF Filho AFS Souza GO Vasconcellos SA Heinemann MB. Immune Response and Protective Profile Elicited by a Multi-Epitope Chimeric Protein Derived From *Leptospira Interrogans* . Int J Inf Dis (2017) 57:61–9. doi: 10.1016/j.ijid.2017.01.032 28161462

[70] Validi M Karkhah A Prajapati VK Nouri HR. Immuno-Informatics Based Approaches to Design a Novel Multi Epitope-Based Vaccine for Immune Response Reinforcement Against Leptospirosis. Mol Immunol (2018) 104:128–38. doi: 10.1016/j.molimm.2018.11.005 30448609

[71] Koizumi N Watanabe H. Molecular Cloning and Characterization of a Novel Leptospiral Lipoprotein With OmpA Domain. FEMS Microbiol Lett (2003) 226(2):215–9. doi: 10.1016/S0378-1097(03)00619-0 14553914

[72] Palaniappan RU McDonough SP Divers TJ Chen CS Pan MJ Matsumoto M. Immunoprotection of Recombinant Leptospiral Immunoglobulin-Like Protein A Against *Leptospira Interrogans* Serovar Pomona Infection. Infect Immun (2006) 74(3):1745–50. doi: 10.1128/IAI.74.3.1745-1750.2006 PMC1418682 16495547

[73] Lee SH Kim S Park SC Kim MJ. Cytotoxic Activities of *Leptospira Interrogans* Hemolysin SphH as a Pore-Forming Protein on Mammalian Cells. Infect Immun (2002) 70(1):315–22. doi: 10.1128/IAI.70.1.315-322.2002 PMC127624 11748197

[74] Flannery B Costa D Carvalho FP Guerreiro H Matsunaga J Da Silva ED. Evaluation of Recombinant *Leptospira* Antigen-Based Enzyme-Linked Immunosorbent Assays for the Serodiagnosis of Leptospirosis. J Clin Microbiol (2001) 39(9):3303–10. doi: 10.1128/JCM.39.9.3303-3310.2001 PMC88335 11526167

[75] Bhasin M Raghava GP. Prediction of CTL Epitopes Using QM, SVM and ANN Techniques. Vaccine (2004) 22(23-24):3195–204. doi: 10.1016/j.vaccine.2004.02.005 15297074

[76] Dhanda SK Gupta S Vir P Raghava GP. Prediction of IL4 Inducing Peptides. Clin Dev Immunol (2013) 2013:263952. doi: 10.1155/2013/263952 24489573 PMC3893860

[77] Garba B Bahaman AR Zakaria Z Bejo SK Mutalib AR Bande F. Antigenic Potential of a Recombinant Polyvalent DNA Vaccine Against Pathogenic Leptospiral Infection. Microb Pathog (2018) 124:136–44. doi: 10.1016/j.micpath.2018.08.028 30138761

[78] Takeda K Akira S. Toll-Like Receptors. Curr Protoc Immunol (2015) 109(1):14.2.1–.2.0. doi: 10.1002/0471142735.im1412s109 25845562

[79] Kim YK Shin JS Nahm MH. NOD-Like Receptors in Infection, Immunity, and Diseases. Yonsei Med J (2016) 57(1):5–14. doi: 10.3349/ymj.2016.57.1.5 26632377 PMC4696971

[80] Werts C Tapping RI Mathison JC Chuang TH Kravchenko V Girons IS. Leptospiral Lipopolysaccharide Activates Cells Through a TLR2-Dependent Mechanism. Nat Immunol (2001) 2(4):346–52. doi: 10.1038/86354 11276206

[81] Nahori MA Fournie-Amazouz E Que-Gewirth NS Balloy V Chignard M Raetz CRH. Differential TLR Recognition of Leptospiral Lipid A and Lipopolysaccharide in Murine and Human Cells. J Immunol (2005) 175(9):6022–31. doi: 10.4049/jimmunol.175.9.6022 16237097

[82] Chassin C Picardeau M Goujon JM Bourhy P Quellard N Darche S. TLR4- and TLR2-Mediated B Cell Responses Control the Clearance of the Bacterial Pathogen, *Leptospira Interrogans* . J Immunol (2009) 183(4):2669–77. doi: 10.4049/jimmunol.0900506 19635914

[83] Holzapfel M Bonhomme D Cagliero J Vernel-Pauillac F Fanton d’Andon M Bortolussi S. Escape of TLR5 Recognition by *Leptospira* Spp.: A Rationale for Atypical Endoflagella. Front Immunol (2020) 11:2007. doi: 10.3389/fimmu.2020.02007 32849665 PMC7431986

[84] Ratet G Santecchia I Fanton d’Andon M Vernel-Pauillac F Wheeler R Lenormand P. LipL21 Lipoprotein Binding to Peptidoglycan Enables *Leptospira Interrogans* to Escape NOD1 and NOD2 Recognition. PLoS Pathog (2017) 13(12):e1006725. doi: 10.1371/journal.ppat.1006725 29211798 PMC5764436

[85] Zhang W Zhang N Xie X Guo J Jin X Xue F. Toll-Like Receptor 2 Agonist Pam3CSK4 Alleviates the Pathology of Leptospirosis in Hamster. Infect Immun (2016) 84(12):3350–7. doi: 10.1128/IAI.00708-16 PMC5116713 27620721

[86] Zhang W Xie X Wang J Song N Lv T Wu D. Increased Inflammation With Crude E. Coli LPS Protects Against Acute Leptospirosis in Hamsters. Emerg Microbes Infect (2020) 9(1):140–7. doi: 10.1080/22221751.2019.1710435 PMC6968624 31914888

[87] Wang J Jin Z Zhang W Xie X Song N Lv T. The Preventable Efficacy of Beta-Glucan Against Leptospirosis. PLoS Negl Trop Dis (2019) 13(11):e0007789. doi: 10.1371/journal.pntd.0007789 31675378 PMC6860453

[88] Goodridge HS Wolf AJ Underhill DM. Beta-Glucan Recognition by the Innate Immune System. Immunol Rev (2009) 230(1):38–50. doi: 10.1111/j.1600-065X.2009.00793.x 19594628 PMC6618291

[89] Potula HH Richer L Werts C Gomes-Solecki M. Pre-Treatment With *Lactobacillus Plantarum* Prevents Severe Pathogenesis in Mice Infected With *Leptospira Interrogans* and May Be Associated With Recruitment of Myeloid Cells. PLoS Negl Trop Dis (2017) 11(8):16. doi: 10.1371/journal.pntd.0005870 PMC5589268 28841659

[90] Liu YW Liong MT Tsai YC. New Perspectives of *Lactobacillus plantarum* as a Probiotic: The Gut-Heart-Brain Axis. J Microbiol (2018) 56(9):601–13. doi: 10.1007/s12275-018-8079-2 30141154

[91] dos Santos TF Melo TA Almeida ME Rezende RP Romano CC. Immunomodulatory Effects of *Lactobacillus plantarum* Lp62 on Intestinal Epithelial and Mononuclear Cells. BioMed Res Int (2016) 2016:8404156. doi: 10.1155/2016/8404156 27446958 PMC4944036

[92] Rakoff-Nahoum S Paglino J Eslami-Varzaneh F Edberg S Medzhitov R. Recognition of Commensal Microflora by Toll-Like Receptors Is Required for Intestinal Homeostasis. Cell (2004) 118(2):229–41. doi: 10.1016/j.cell.2004.07.002 15260992

[93] Koizumi S Wakita D Sato T Mitamura R Izumo T Shibata H. Essential Role of Toll-Like Receptors for Dendritic Cell and NK1.1(+) Cell-Dependent Activation of Type 1 Immunity by *Lactobacillus pentosus* Strain S-Pt84. Immunol Lett (2008) 120(1-2):14–9. doi: 10.1016/j.imlet.2008.06.003 18620001

[94] Liu Y Fatheree NY Mangalat N Rhoads JM. *Lactobacillus reuteri* Strains Reduce Incidence and Severity of Experimental Necrotizing Enterocolitis *via* Modulation of TLR4 and NF-kB Signaling in the Intestine. Am J Physiol Gastrointest Liver Physiol (2012) 302(6):G608–17. doi: 10.1152/ajpgi.00266.2011 PMC3311308 22207578

[95] Wu Q Liu MC Yang J Wang JF Zhu YH. *Lactobacillus Rhamnosus* GR-1 Ameliorates *Escherichia coli*-Induced Inflammation and Cell Damage *via* Attenuation of ASC-Independent NLRP3 Inflammasome Activation. Appl Environ Microbiol (2016) 82(4):1173–82. doi: 10.1128/AEM.03044-15 PMC4751844 26655757

[96] Gabryszewski SJ Bachar O Dyer KD Percopo CM Killoran KE Domachowske JB. *Lactobacillus-*Mediated Priming of the Respiratory Mucosa Protects Against Lethal Pneumovirus Infection. J Immunol (2011) 186(2):1151–61. doi: 10.4049/jimmunol.1001751 PMC3404433 21169550

[97] Garcia-Crespo KE Chan CC Gabryszewski SJ Percopo CM Rigaux P Dyer KD. *Lactobacillus* Priming of the Respiratory Tract: Heterologous Immunity and Protection Against Lethal Pneumovirus Infection. Antiviral Res (2013) 97(3):270–9. doi: 10.1016/j.antiviral.2012.12.022 PMC3608699 23274789

[98] Rice TA Brenner TA Percopo CM Ma M Keicher JD Domachowske JB. Signaling *via* Pattern Recognition Receptors NOD2 and TLR2 Contributes to Immunomodulatory Control of Lethal Pneumovirus Infection. Antiviral Res (2016) 132:131–40. doi: 10.1016/j.antiviral.2016.06.002 PMC4980231 27312104

[99] Santecchia I Vernel-Pauillac F Rasid O Quintin J Gomes-Solecki M Boneca IG. Innate Immune Memory Through TLR2 and NOD2 Contributes to the Control of *Leptospira Interrogans* Infection. PLoS Pathog (2019) 15(5):26. doi: 10.1371/journal.ppat.1007811 PMC6544334 31107928

[100] Nascimento AL Ko AI Martins EA Monteiro-Vitorello CB Ho PL Haake DA. Comparative Genomics of Two *Leptospira Interrogans* Serovars Reveals Novel Insights Into Physiology and Pathogenesis. J Bacteriol (2004) 186(7):2164–72. doi: 10.1128/JB.186.7.2164-2172.2004 PMC374407 15028702

[101] Gamberini M Gómez RM Atzingen MV Martins EA Vasconcellos SA Romero EC. Whole-Genome Analysis of *Leptospira Interrogans* to Identify Potential Vaccine Candidates Against Leptospirosis. FEMS Microbiol Lett (2005) 244(2):305–13. doi: 10.1016/j.femsle.2005.02.004 15766783

[102] Dellagostin OA Grassmann AA Hartwig DD Félix SR da Silva É McBride AJ. Recombinant Vaccines Against Leptospirosis. Hum Vaccin (2011) 7(11):1215–24. doi: 10.4161/hv.7.11.17944 22048111

[103] Atzingen MV Vieira ML Oliveira R Domingos RF Mendes RS Barros AT. Evaluation of Immunoprotective Activity of Six Leptospiral Proteins in the Hamster Model of Leptospirosis. Open Microbiol J (2012) 6:79–87. doi: 10.2174/1874285801206010079 23173023 PMC3502890

[104] Jackson NAC Kester KE Casimiro D Gurunathan S DeRosa F. The Promise of mRNA Vaccines: A Biotech and Industrial Perspective. NPJ Vaccines (2020) 5(1):11. doi: 10.1038/s41541-020-0159-8 32047656 PMC7000814

[105] Pardi N Hogan MJ Porter FW Weissman D. mRNA Vaccines - a New Era in Vaccinology. Nat Rev Drug Discov (2018) 17(4):261–79. doi: 10.1038/nrd.2017.243 PMC5906799 29326426

[106] Chen N Xia P Li S Zhang T Wang TT Zhu J. RNA Sensors of the Innate Immune System and Their Detection of Pathogens. IUBMB Life (2017) 69(5):297–304. doi: 10.1002/iub.1625 28374903 PMC7165898

[107] Maine CJ Richard G Spasova DS Miyake-Stoner SJ Sparks J Moise L. Self-Replicating RNAs Drive Protective Anti-Tumor T Cell Responses to Neoantigen Vaccine Targets in a Combinatorial Approach. Mol Ther (2020) 29(3):1186–98. doi: 10.1016/j.ymthe.2020.11.027 PMC7934630 33278563

[108] Szurgot I Ljungberg K Kümmerer BM Liljeström P. Infectious RNA Vaccine Protects Mice Against Chikungunya Virus Infection. Sci Rep (2020) 10(1):21076. doi: 10.1038/s41598-020-78009-7 33273501 PMC7712826

[109] Pardi N Hogan MJ Pelc RS Muramatsu H Andersen H DeMaso CR. Zika Virus Protection by a Single Low-Dose Nucleoside-Modified mRNA Vaccination. Nature (2017) 543(7644):248–51. doi: 10.1038/nature21428 PMC5344708 28151488

[110] Polack FP Thomas SJ Kitchin N Absalon J Gurtman A Lockhart S. Safety and Efficacy of the BNT162b2 mRNA Covid-19 Vaccine. N Engl J Med (2020) 383(27):2603–15. doi: 10.1056/NEJMoa2034577 PMC7745181 33301246

[111] Baden LR El Sahly HM Essink B Kotloff K Frey S Novak R. Efficacy and Safety of the mRNA-1273 SARS-CoV-2 Vaccine. N Engl J Med (2021) 384(5):403–16. doi: 10.1056/NEJMoa2035389 PMC7787219 33378609

